# Modified Neumann incision for implant placement in overdentures: Case series of a less invasive alternative

**DOI:** 10.1002/cap.70044

**Published:** 2026-03-23

**Authors:** Lucas Jardim da Silva, Laura Lourenço Morel, Luciana de Rezende Pinto, Otacílio Luiz Chagas‐Júnior, Jamil Awad Shibli, Fernanda Faot

**Affiliations:** ^1^ Graduate Program in Dentistry School of Dentistry Federal University of Pelotas (UFPel) Pelotas Rio Grande do Sul Brazil; ^2^ Department of Restorative Dentistry School of Dentistry Federal University of Pelotas (UFPel) Pelotas Rio Grande do Sul Brazil; ^3^ Department of Oral and Maxillofacial Surgery and Maxillofacial Prosthodontics School of Dentistry Federal University of Pelotas (UFPel) Pelotas Rio Grande do Sul Brazil; ^4^ Dental Research Division Guarulhos University Guarulhos São Paulo Brazil; ^5^ Albert Einstein Israeli Faculty of Health Sciences Albert Einstein Israelite Hospital São Paulo Brazil; ^6^ Department of Oral Medicine, Infection and Immunity Harvard School of Dental Medicine Boston USA; ^7^ Department of Oral Health Sciences Periodontology and Oral Microbiology KU Leuven Belgium

**Keywords:** case series, dental implants, digital planning, intervention, minimally invasive surgery, surgical technique

## Abstract

**Background:**

Conventional envelope flap techniques for implant placement in edentulous mandibles are associated with greater postoperative morbidity, especially in elderly patients or those with anatomical limitations. Minimally invasive alternatives aim to preserve soft tissues and reduce complications, enhancing clinical predictability.

**Methods:**

A clinical study was conducted with ten fully edentulous patients, in whom 20 regular implants were placed in the interforaminal region without surgical guides. A modified Neumann incision with a mesial relaxing cut and partial‐thickness “L”‐shaped flap was used. Digital planning was performed using free software. Surgical time, number of anesthetic tubes, adverse events, flap healing, peri‐implant inflammation (PI), gingival bleeding index (GBI), and probing depth (PD) were evaluated at 14, 30, 60, and 90 days postoperatively.

**Results:**

No surgical complications, such as dehiscence, infection, paresthesia, or early implant loss, were observed. All patients achieved primary wound healing. The mean surgical time was 44 min (range: 25–61 min), with an average of four anesthetic tubes per patient. GBI and PD remained low throughout follow‐up, with few sites showing PD ≥ 4 mm. Healing scores and inflammatory parameters progressively improved over time.

**Conclusions:**

The modified Neumann incision with mesial relaxing cut, guided by digital planning, proved to be a safe, predictable, and minimally invasive approach for implant placement in edentulous mandibles. This technique demonstrated favorable healing, reduced surgical time, and effective PI control, making it suitable for patients with systemic fragility or anatomical challenges.

**Key points:**

- The modified Neumann incision with mesial relaxing cut allows for safe and direct visualization of the mental foramen with limited flap elevation, reducing surgical trauma and preserving soft tissue integrity.
- This technique demonstrated favorable healing outcomes and low postoperative morbidity, even in elderly patients and anatomically challenging mandibles, without the need for physical surgical guides.
- Digital preoperative planning using free software enhances precision and predictability, making the approach reproducible and applicable in daily clinical practice.

**Plain language summary:**

This study describes a new, less invasive surgical technique for placing dental implants in the lower jaw of patients who have lost all their teeth. The method, called the modified Neumann incision with a mesial relaxing cut, was tested in 10 patients who received 2 implants to support a mandibular overdenture. The technique allows the surgeon to see important anatomical structures, such as nerves, while reducing the amount of tissue that needs to be cut or lifted. Patients were followed for 3 months after surgery, and the healing process, inflammation, and peri‐implant health were monitored. The results showed that all patients healed well, with no significant complications or pain, and the soft tissues around the implants remained healthy. The approach proved to be safe, efficient, and well tolerated, particularly for older adults and individuals with health conditions that increase surgical risks. This technique may help make implant treatments more comfortable and predictable for edentulous patients, although larger studies are still needed to confirm these findings.

## INTRODUCTION

Two narrow implants placement in the anterior region of the mandible to retain overdentures is a well‐established approach in the edentulous patient rehabilitation (Liu et al., 2025).^1^, ^2^, ^3^, ^4^ This strategy has proven effective in improving masticatory function, prosthetic stability, and quality of life, especially in elderly individuals, regardless of the loading protocol, as demonstrated in randomized clinical trials.^5^, ^6^

Traditionally, the most used surgical technique for this procedure is the envelope‐type incision, involving wide mucosal detachment and exposure of the edentulous ridge. However, this approach presents significant clinical limitations, especially in older patients—who represent the majority of those undergoing this type of rehabilitation.^7^ The conventional technique requires a broader surgical field, with extensive detachment of the mucosa and adjacent musculature, which can result in greater postoperative morbidity, such as pain, swelling, bleeding, and a higher risk of suture dehiscence. These factors may compromise healing and the initial stability of the implants, in addition to negatively impacting the patient's clinical experience.^8^

Considering the inherent limitations of conventional approaches, several surgical techniques have been proposed to minimize tissue trauma and promote a more conservative procedure. The Neumann incision, originally described in 1982, consists of a lateral linear approach to the midline with minimal flap detachment, allowing the adequate visualization of the alveolar crest while preserving vascular supply and potentially reducing postoperative recovery time. In the present study, a modified L‐shaped Neumann incision is proposed.^9^ This modification avoids crossing the mandibular midline and incorporates an “L”‐shaped relaxing incision, enabling frontal and direct visualization of the mental foramen after mucosal detachment. The technique ensures optimal access to the interforaminal region with reduced soft tissue trauma while being anatomically guided to respect natural tissue boundaries and preserve the integrity of surrounding structures.

The clinical applicability of this modified approach is strongly supported by contemporary digital technology, which enables accurate pre‐visualization of critical anatomical structures. Through digital planning software—a freely accessible platform—three‐dimensional computed tomography data can be analyzed to precisely identify the mental foramen, trace the mandibular canal trajectory, and evaluate the bone morphology of the interforaminal region. This digitally driven preoperative mapping enhances accuracy, predictability, and safety, allowing for more targeted incisions and optimal tissue management, even in the absence of a physical surgical guide.^10^

Therefore, the objective of this technical report is to present, step by step, the execution of the modified “L”‐shaped Neumann incision, demonstrating its clinical feasibility as a minimally invasive alternative for implant placement in edentulous mandibles in the context of overdenture rehabilitation. This case series has been reported in line with the PROCESS guidelines.^11^

## MATERIALS AND METHODS

### Study design

The surgical technique described in this report was employed within the context of a randomized, prospective, and parallel clinical trial conducted in accordance with the CONSORT (Consolidated Standards of Reporting Trials) guidelines for controlled clinical studies.^12^ The study was approved by the Human Research Ethics Committee of the Federal University of Pelotas (protocol 6.420.934) and conducted between the years 2023 and 2025. This study was conducted in accordance with the principles of the Declaration of Helsinki and was approved by the Human Research Ethics Committee of the Federal University of Pelotas (protocol 6.420.934) and conducted between the years 2023 and 2025. This 10‐case series report was prepared in accordance with the PROCESS 2020 checklist for case series.^13^ All patients signed the informed consent form approved by the institutional ethics committee.

Ten fully edentulous patients were included, all of whom received two conventional complete dentures (CCD) (upper and lower) and were rehabilitated with two regular‐diameter implants (*Ø*3.5 × 10 mm, Plenum Bioengenharia) produced by additive manufacturing. Treatment was carried out at the Complete Denture Clinic of the School of Dentistry at the Federal University of Pelotas (UFPel), and the implants were placed exclusively using the modified Neumann incision technique proposed in this study.

Participants were allocated to either the immediate or conventional loading group through a simple random draw (sealed envelope method) conducted immediately after implant installation. However, to be eligible for the immediate loading group, patients were required to achieve a minimum insertion torque of 32 N cm in both implants. When this mechanical stability threshold was not reached, the patient was automatically assigned to the conventional loading group.

Inclusion criteria comprised fully edentulous patients wearing complete dentures in both arches for at least 3 months, with medically controlled systemic conditions such as hypertension and diabetes, and with adequate bone availability for implant placement without the need for grafts. Smokers, patients with severe systemic diseases, bisphosphonate use, a history of head and neck radiotherapy, immunosuppression, pregnancy, or breastfeeding, among other exclusion factors, were excluded from the sample.

### Description of the surgical technique

The proposed technique is based on the modified Neumann incision, performed with an “L”‐shaped relaxing cut at the mesial angle and restricted to each hemimandible, without crossing the midline. This approach allows for a partial‐thickness flap elevation for direct visualization of the mental foramen with minimal tissue trauma, enabling the placement of interforaminal implants with greater anatomical precision.

The flap elevation is limited to the area previously defined during digital planning, preserving the adjacent soft tissues and reducing the operative field. The incision is made linearly along the crest of the ridge with a vertical releasing cut, ensuring frontal access to the foramen and adequate exposure of the alveolar crest.

### Digital planning

**FIGURE 1:**
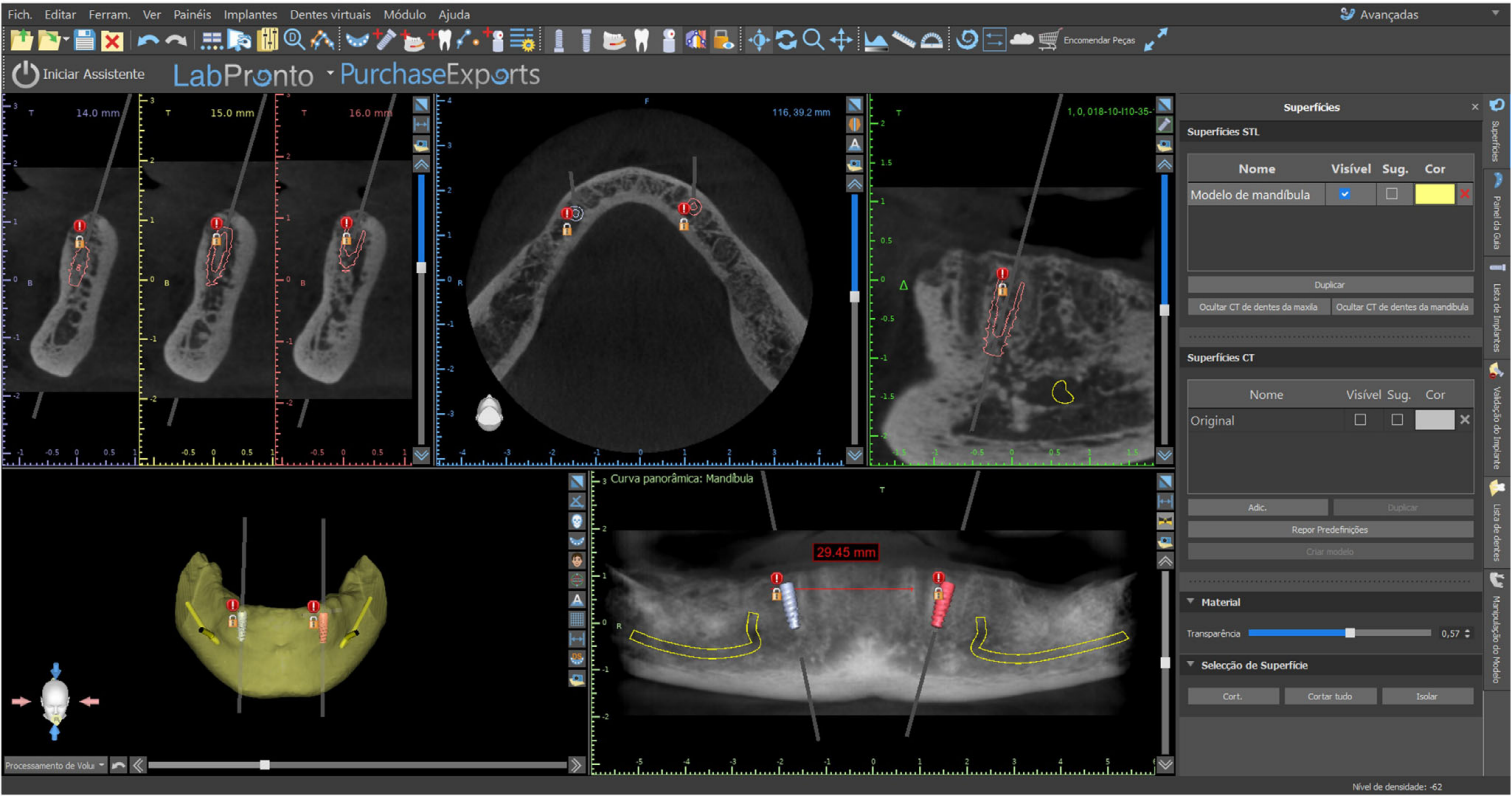
Planning performed using Blue Sky Plan software (Libertyville, IL, USA).

Surgical planning was carried out in advance using special software (Blue Sky Plan, Libertyville, IL, USA) (Figure 1), on the basis of cone‐beam computed tomography (Eagle Edge 0.2 FS, Dabi Atlante). The goal was to accurately determine the position of the mental foramina, available bone thickness, trajectory of the mandibular canal, and the ideal distance between implants.

**FIGURE 2:**
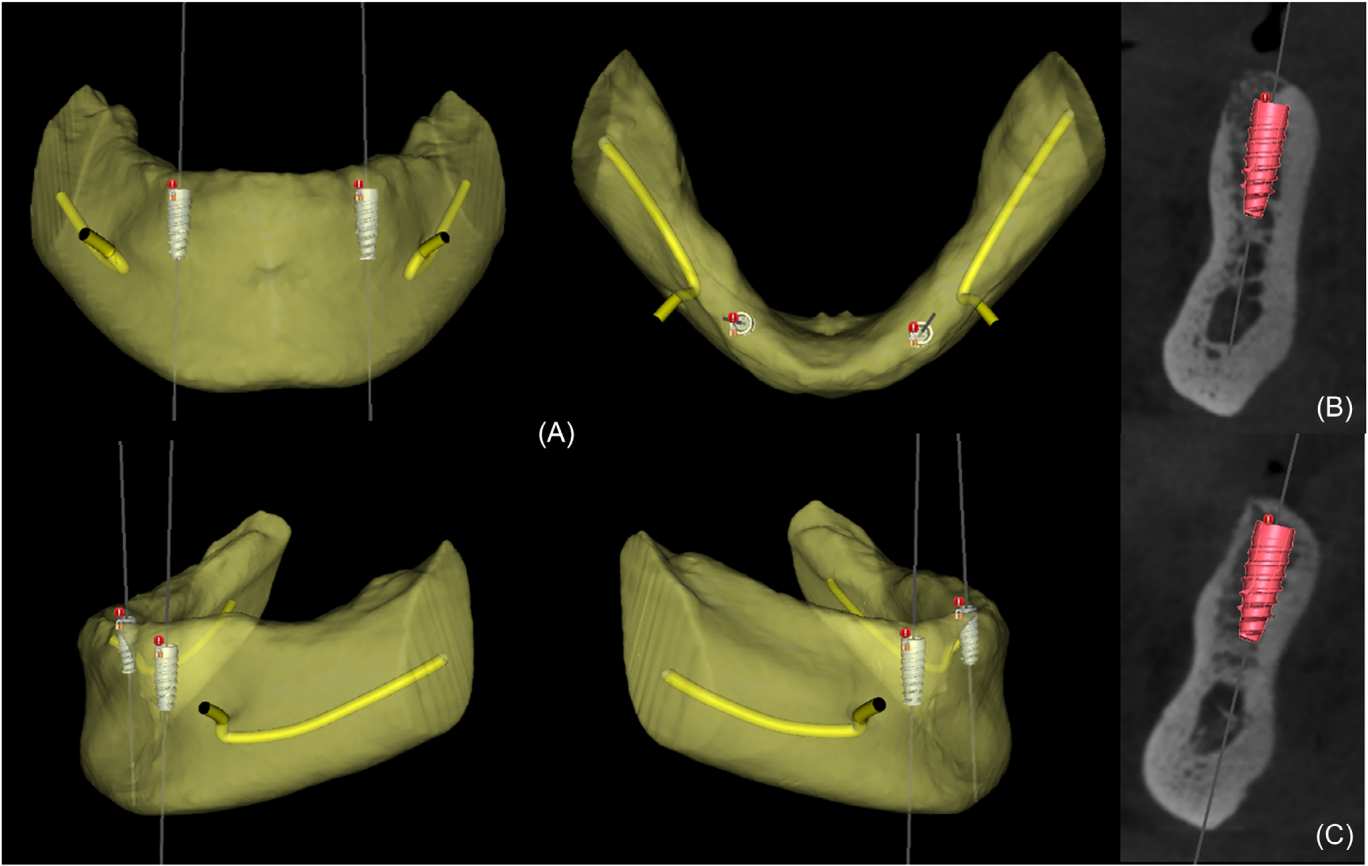
(A) 3D reconstruction of the mandible with implant planning. (B) Sagittal view of the implant planning on the right side. (C) Sagittal view of the implant planning on the left side.

The three‐dimensional visualization of anatomical structures enabled the safe definition of the incision site and implant positions, ensuring predictability for the proposed technique (Figure 2). It is important to note that no physical surgical guide was used during the procedures. Implant positioning was guided solely by the anatomical references obtained from the virtual planning.

### Surgical procedure

A standardized one‐stage surgical protocol was performed by an experienced surgeon (OLCJ) for the placement of all implants. As preoperative medication, 40 mg of prednisolone was prescribed and administered 1 h prior to the procedure to reduce the postoperative inflammatory response.

Antisepsis was performed using 0.12% chlorhexidine digluconate intraorally and 2% extraorally. The patient was then anesthetized via vestibular and lingual infiltration at the depth of the sulcus, using 4% articaine with 1:100,000 epinephrine (DFL Indústria e Comércio S/A).

**FIGURE 3:**
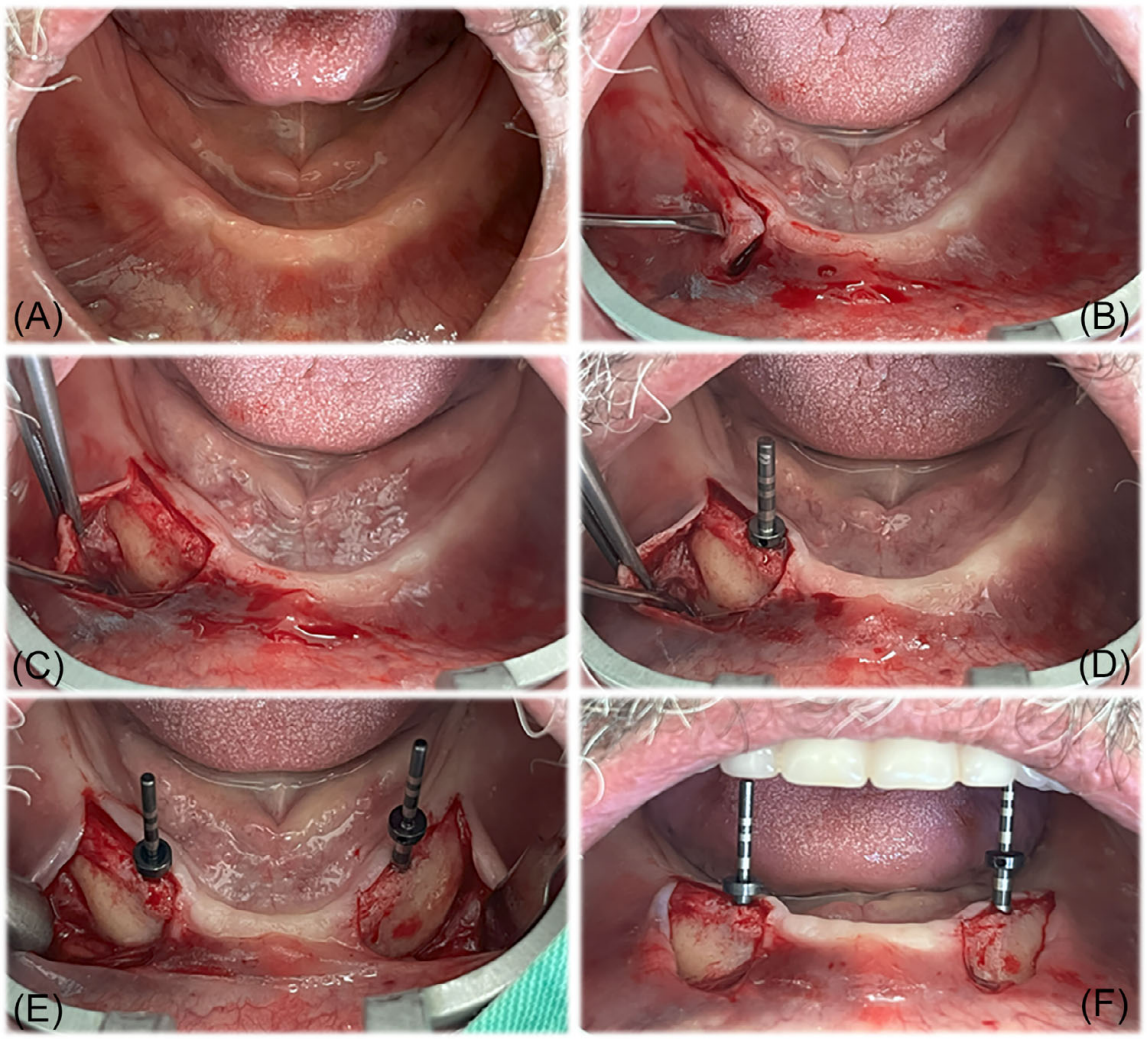
Surgical sequence illustrating the new incision approach: (A) initial ridge; (B) L‐shaped incision; (C) elevation of the mucoperiosteal flap; (D) parallel pin positioned on the right side; (E) parallel pins positioned on both right and left sides; (F) frontal view of the parallel pins, indicating correct mesiodistal positioning of the osteotomy.

The proposed incision technique began with the execution of full‐thickness “L”‐shaped flaps, with the relaxing incision directed towards the mesial region, without crossing the mandibular midline. Each flap was elevated as a limited mucoperiosteal reflection to allow direct visualization of the alveolar crest and the mental foramen, as shown in Figure 3.

This approach provided adequate surgical access with minimal flap extension, enabling clear visualization of critical anatomical structures while preserving the integrity of adjacent soft tissues. With the alveolar crest exposed, osteotomy sites were marked and performed, guided by the distal face of the upper complete denture's lateral incisors, used as a clinical reference. The initial drilling was carried out approximately 5 mm anterior to the mental foramina, maintaining a minimum distance of 20 mm between the two implants, ensuring anatomical safety and proper positioning for future prosthetic installation.

**FIGURE 4:**
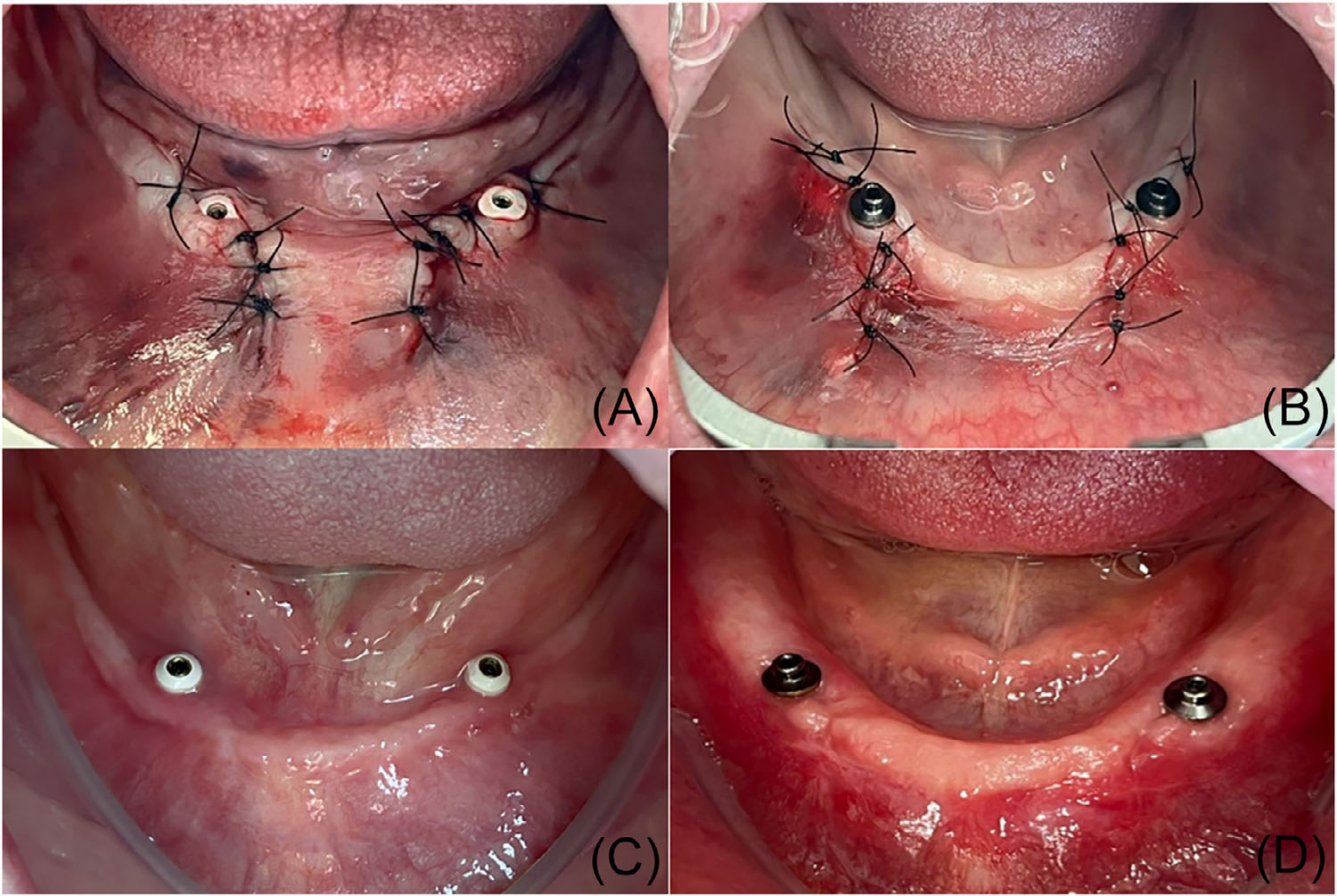
Final aspect after placement of the healing abutment and Equator component, and completion of suturing. (A) Peek healing cap (CL); (B) Equator abutment (IL). Ninety‐day follow‐up. (C) Patient from the conventional loading (CL) group with healing abutment. (D) Patient from the immediate loading (IL) group with Equator.

Finally, the healing abutment or the Equator prosthetic component was connected to the installed implants. The mucoperiosteal flap was then carefully adapted and sutured with simple interrupted stitches, ensuring that the transmucosal components remained fully exposed, avoiding submersion during the healing period (Figure 4).

#### Postoperative examinations, care, and medications

After the procedure, the patient was instructed to undergo a panoramic radiograph to monitor marginal bone loss and was advised to follow the prescribed medication regimen correctly. Instructions included: do not spit; do not blow the nose; sneeze with the mouth open; consume cold liquid or soft foods during the first 3 days; gradually increase food consistency starting on the fourth day; avoid physical exertion until suture (stitches) removal; avoid sun exposure for 15 days; do not drink hot liquids for 7 days; avoid rinsing the mouth for 15 days; brush teeth normally but carefully around the surgical area (without rinsing); remain seated and use a slightly elevated pillow when sleeping; minimize talking—speak only when necessary; avoid touching the surgical site with the tongue.

For patients who received immediate loading, they were instructed not to remove the prosthesis during the first 48 h to help control swelling and to perform hygiene by flushing under the overdenture with a 20 mL syringe and saline solution.

Postoperative medication included 500 mg amoxicillin three times a day for 7 days, 600 mg ibuprofen three times a day for 4 days, and 500 mg paracetamol four times a day for 3 days. Nylon sutures (Procare; Lamedid Ltda) were removed 10 days after surgery. Comprehensive postoperative and prosthesis care instructions were provided to all patients and reinforced at each follow‐up visit.

#### Clinical evaluation criteria

Peri‐implant inflammation (PI) and surgical flap healing were evaluated at 7, 14, 30, 60, and 90 days postoperatively. PI was assessed through visual inspection of the peri‐implant mucosa, considering the presence of redness, edema, or bleeding upon gentle probing. Inflammation was recorded as “1” when any of these signs were present and “0” when absent.

Flap healing was evaluated by visual inspection of the mucosal surface according to the criteria described by Wachtel et al.^14^ and categorized as follows: (1) completely healed, characterized by intact mucosa and absence of fibrin or necrosis; (2) partial healing, with the presence of fibrin; and (3) compromised healing, exhibiting necrosis or tissue dehiscence.

Peri‐implant health was assessed following the method proposed by Mombelli et al.,^15^ including evaluation of the Visible Plaque Index (VPI), Peri‐implant Inflammation (PI), Calculus Presence (CP), probing depth (PD), and bleeding on probing (BOP). Clinical examinations were performed at 7, 14, 30, 60, and 90 days postoperatively on four surfaces of each implant (mesial, distal, buccal, and lingual).

#### Timeline

Initially, all patients were rehabilitated with CCD for both the maxilla and mandible and underwent a 3‐month adaptation period. After this period, two implants were placed in the interforaminal region of the mandible. Patients who presented an insertion torque equal to or greater than 32 N cm were subjected to immediate loading, with the conversion of their lower dentures into mandibular overdentures on the same day of surgery. Patients whose insertion torque was below 32 N cm followed the conventional loading protocol, waiting 3 months before prosthesis conversion into overdentures.

Clinical follow‐up was carried out at the following stages:

- 7 days: evaluation of inflammation and healing of the surgical flap;
- 10 days: suture removal;
- 14 days: reassessment of inflammation, flap healing, and peri‐implant health;
- 30 days: reassessment of inflammation, flap healing, and peri‐implant health;
- 60 days: reassessment of inflammation, flap healing, and peri‐implant health;
- 90 days: reassessment of inflammation, flap healing, and peri‐implant health.

At all follow‐up visits, the presence of extraoral or intraoral edema, signs of sensitivity, and reported levels of pain were recorded through directed anamnesis (without the use of a VAS scale).

## RESULTS

The sample consisted of 10 fully edentulous patients, divided between the immediate loading group (*n* = 6) and the conventional loading group (*n* = 4). The mean age was 67.0 ± 8.41 years, with patients in the conventional loading group being older (73.8 ± 5.56 years) compared to those in the immediate loading group (62.5 ± 6.92 years). The mean duration of mandibular edentulism was 8.6 ± 13.92 years, ranging from 0 to 44 years, with an average of 5.2 years in the immediate loading group and 13.8 years in the conventional group. Sample characterization is shown in Table 1.

**TABLE 1:** Sample characterization.

Variable	IL (*n* = 6)	CL (*n* = 4)	Total (*n* = 10)
Age (years)	62.5 ± 6.92	73.8 ± 5.56	67.0 ± 8.41
Edentulism duration (years)	5.2 ± 7.57	13.8 ± 20.66	8.6 ± 13.92
Bone regularization (Yes/No)	3/3	1/3	4/6
Mandibular atrophy (Yes/No)	1/5	0/4	1/9
Ridge crest—Plateau	2	1	3
Ridge crest—Knife‐edge	2	3	5
Ridge crest—Regular	1	1	2
Bone type—Type I	3	4	7
Bone type—Type II	3	0	3

Regarding mandibular anatomical characteristics, the most observed ridge shapes were knife‐edge (*n* = 5), plateau (*n* = 3), and regular ridge (*n* = 2), with similar distribution between the groups. Most patients presented with bone type I (*n* = 7), whereas type II was found in three patients, all from the immediate loading group. The need for bone regularization was recorded in four patients (three from the immediate group and one from the conventional group), performed solely to create a flat platform and prevent drill slippage during osteotomy. Only one case presented with mandibular atrophy, which belonged to the immediate loading group.

All patients underwent the placement of two implants between the mental foramina, totaling 20 implants installed using the modified Neumann incision technique with a mesial relaxing cut. The technique was applied in six immediate loading cases and four conventional loading cases, demonstrating its applicability across different clinical approaches. All procedures were based on prior virtual planning and executed without the use of a physical surgical guide. The incision allowed frontal and direct visualization of the mental foramen, with limited flap elevation and safe anatomical access, favoring precise implant placement. All implants were installed at bone level, 2 mm below the alveolar crest. The average surgical time was approximately 44 min, varying according to the complexity of each case. In all cases, four cartridges of 4% articaine with 1:100,000 epinephrine (DFL) were used.

In clinical outcomes as shown in Table 2, PI was present in all cases during the early stages (7–60 days) in both groups, with a progressive decrease toward the end of the follow‐up. By Day 90, inflammation was absent in all cases of the conventional group and persisted in only 58.3% of the immediate group. Healing of the surgical flap showed a parallel improvement trend, with partial healing (presence of fibrin) and necrosis predominating during the early stages but complete resolution in all implants by Day 90. The immediate loading group demonstrated slightly faster recovery, with a higher percentage of complete healing (41.7) already observed by 60 days compared to the conventional group (25).

**TABLE 2:** Time‐based progression of inflammation and healing of implants in the immediate and conventional loading group.

Group	Time (days)	Inflammation present (%)	Complete healing (%)	Partial healing with fibrin (%)	Necrosis (%)
Immediate	7	100	0	58.33	33.33
	14	100	0	75	25
	30	100	16.66	41.66	41.66
	60	100	41.66	25	33.33
	90	58.33	100	0	0
Conventional	7	100	0	25	75
	14	100	0	50	50
	30	100	25	50	25
	60	100	25	50	25
	90	50	100	0	0

According to Table 3, gingival bleeding index (GBI) scores also revealed a progressive improvement throughout the 90‐day follow‐up. Bleeding was observed in over 90% of implants at the initial assessments (14–30 days) in both groups, but by Day 90, the bleeding rate had dropped to 33.3% in the immediate group and 75% in the conventional group. This progressive decline suggests effective control of PI, particularly in the immediate loading group.

**TABLE 3:** Gingival bleeding index (GBI)—percentage of implants with bleeding.

Time (days)	Immediate loading (%)	Conventional loading (%)
14	91.66	100
30	100	87.5
60	66.66	87.5
90	33.33	75

Mean PD values are presented in Table 4. In both groups, a consistent reduction in PD was observed over time. At 14 days, the immediate group exhibited a mean of 3.2 ± 0.6 mm, compared to 3.7 ± 0.7 mm in the conventional group. By Day 90, these values had decreased to 2.2 ± 0.7 mm and 2.7 ± 0.8 mm, respectively. The downward trend indicates progressive improvement in peri‐implant soft tissue adaptation and stability of the mucosal seal.

**TABLE 4:** Mean probing depth (mm) of implants in the immediate and conventional loading groups at 14, 30, 60, and 90 days.

Time (days)	Immediate loading (mean ± SD)	Conventional loading (mean ± SD)
14	3.2 ± 0.6	3.7 ± 0.7
30	2.4 ± 0.8	2.8 ± 0.7
60	2.1 ± 0.6	2.3 ± 0.1
90	2.2 ± 0.7	2.7 ± 0.8

*Note*: Values represent the mean ± standard deviation (SD) calculated from measurements taken at four sites per implant (mesial, distal, buccal, and lingual).

As detailed in Table 5, the number of sites with PD ≥ 4 mm decreased substantially throughout the study. In the immediate loading group, these sites were relatively frequent at 14 days but nearly absent by Day 60. In the conventional group, a similar pattern was observed, with an initial concentration of deep sites during the first 30 days and almost complete resolution by Day 90.

**TABLE 5:** Number of surfaces with probing depth ≥ 4 mm in implants from the immediate and conventional loading groups at 14, 30, 60, and 90 days.

	14 days		30 days		60 days		90 days	
Group	Right implant	Left implant	Right implant	Left implant	Right implant	Left implant	Right implant	Left implant
Immediate	2	2	0	0	0	0	0	0
	2	2	2	0	0	0	0	1
	1	0	0	0	0	0	0	0
	3	2	0	0	0	0	0	0
	0	4	0	1	0	0	0	0
	2	1	1	1	0	0	0	0
Conventional	4	3	0	0	0	0	3	2
	4	4	3	2	0	0	0	0
	0	1	0	0	0	0	0	0
	0	4	0	2	0	0	0	0

*Note*: The count of probing depths ≥4 mm represents the total number of surfaces with increased depth at each evaluated time point.

Overall, both groups demonstrated satisfactory clinical progression, with steady improvement in peri‐implant conditions over time, evidenced by reduced PDs and fewer sites with depths ≥ 4 mm. These findings indicate effective inflammatory control and maintenance of tissue health around the implants, regardless of the loading protocol adopted. No implant loss was recorded during the follow‐up period, nor were there complications such as paresthesia, infection, hematoma, or suture dehiscence. All patients showed proper primary healing without the need for additional analgesic medication. Spontaneous reports indicated a high level of satisfaction with the procedure, particularly regarding postoperative comfort, absence of significant pain, and rapid recovery.

Most patients expressed enthusiasm about prosthetic rehabilitation and considered the minimally invasive approach an important differentiator compared to their previous dental experiences with CCD. Throughout their lives, these individuals had undergone multiple tooth extractions until reaching complete edentulism, often associating dental treatment with pain and discomfort. Although none of the participants had previously undergone implant surgeries, several reported that they had expected a more traumatic postoperative course based on acquaintances’ experiences with traditional envelope flap procedures. Instead, they described this approach as more comfortable, with faster recovery and minimal postoperative discomfort.

## DISCUSSION

The initial characterization of the sample revealed that the proposed technique was successfully applied in patients with different anatomical profiles and varying degrees of mandibular atrophy, with ridge regularization required in some cases to provide a flat and stable base for the surgical drills. The ability to adapt the technique to different bone morphologies without resorting to extensive incisions reinforces its clinical versatility.

During the postoperative period, no complications, such as dehiscence, paresthesia, infection, or early implant loss, were recorded, and all patients exhibited favorable primary healing. The placement of implants in the interforaminal region of the mandible using the conventional envelope‐type incision technique has been widely employed in overdenture rehabilitations. Randomized clinical trials, such as those conducted by Schuster et al.^5^ and Possebon,^6^ demonstrate that this approach offers high success rates, prosthetic and peri‐implant tissue stability,^16^ and functional improvement in edentulous patients, especially those receiving narrow‐diameter implants using button‐type retention systems (Equator Attachment).

However, the traditional technique requires extensive detachment of the mucosa and musculature, resulting in greater postoperative morbidity. This aspect is particularly relevant in atrophic mandibles, which are frequently associated with complications such as paresthesia and suture dehiscence, compared to areas with preserved bone volume. In such situations, minimally invasive techniques may be advantageous by reducing surgical trauma and preserving the vascularization of soft tissues. Studies have shown a high incidence of altered sensitivity, with up to 13% of cases following conventional mandibular implant surgery, as evidenced by Lin et al.^17^ In atrophic mandibles, the proximity of the neurovascular bundle and the reduced bone thickness increase susceptibility to complications such as nerve injury. Minimally invasive techniques with reduced or transgingival flaps have been shown to reduce these risks by preserving local morphology and blood supply, as described by Jeong et al.^8^

The results related to flap healing and PI demonstrated favorable tissue responses over time, even with the adoption of immediate loading. The immediate loading group presented favorable healing scores from the early stages, with complete resolution in all cases by 90 days. Inflammation also progressively decreased, with minimal or absent presence at the end of the period. The favorable inflammatory and healing response supports the safety of the approach even under greater initial functional demand, as seen in immediate loading protocols.^18^, ^19^

Monitoring peri‐implant health using the GBI and PD Index supported these findings. Although GBI revealed bleeding in most cases during the early period, especially in the conventional group, this condition was controlled over time. Meanwhile, PD demonstrated peri‐implant tissue stability, with mean depths below 3 mm at all timepoints and few sites with PD ≥ 4 mm, indicating no significant peri‐implant compromise. These findings are consistent with the literature, which links good surgical technique and reduced trauma to improved peri‐implant health.^20^, ^21^

The preservation of keratinized mucosa was consistently observed during the healing process in all cases. As the incision design was restricted to the alveolar crest and avoided excessive vestibular displacement, the band of keratinized tissue surrounding the healing abutments remained stable throughout the follow‐up. This finding is clinically relevant, as adequate keratinized mucosa has been associated with improved peri‐implant health, reduced mucosal inflammation, and enhanced patient comfort during oral hygiene procedures.^22^, ^23^ The maintenance of this soft‐tissue stability supports the minimally invasive concept of the modified Neumann incision, which aims to preserve vascularization and mucosal continuity in the interforaminal region.

The modified Neumann incision technique with mesial relaxing cut proposed in this study proved to be an effective and less invasive alternative, allowing safe exposure of the alveolar crest with limited flap elevation. Direct visualization of the mental foramen provides greater control of the region and reduces the risk of neurosensory complications—which is particularly important in patients with critical anatomy. From an anatomical and functional standpoint, it is worth noting that the genial tubercles (geni) and the insertion of the mentalis muscle are located closer to the crest in the anterior mandibular region. The proposed technique enables preservation of these structures, avoiding their detachment and, consequently, the risks associated with failed reinsertion. This preservation is directly related to the reduced occurrence of dehiscences and better prosthesis adaptation in the postoperative period. Furthermore, the limited detachment of the lower labial musculature appears to have contributed to the lower incidence of edema observed in the operated patients. It is important to highlight that, in conventional approaches involving broad detachment of vestibular and lingual musculature in the midline region of the mandible, there is a tendency for elevation of the mouth floor—a situation not observed with the technique described here, reinforcing its less traumatic and potentially safer nature.

Another key factor in the success of the technique was the use of the free implant planning software, which allowed precise three‐dimensional planning even without the use of physical surgical guides. The literature supports that virtual planning based on CBCT improves implant placement accuracy, reduces surgical time, and increases clinical predictability even in techniques without printed guides, as suggested by Azevedo et al. (2024)^10^ and Pereira et al.^24^ In addition, conservative techniques such as mini‐incisions and transgingival approaches have demonstrated similar efficacy to full‐thickness flap surgeries in terms of osseointegration, with the added benefits of lower tissue morbidity, vascular preservation, and more comfortable recovery for the patient, as emphasized by Jeong et al.^8^ However, it is important to acknowledge that the described technique is not indicated for all cases. In extremely atrophic mandibles requiring extensive osteoplasty, the limited access provided by the proposed incision may be insufficient, making the conventional incision approach more appropriate.

In summary, the modified Neumann incision with mesial relaxing cut represents a safe, predictable, and less invasive alternative for the rehabilitation of edentulous mandibles with overdentures. Its benefits are particularly relevant for elderly patients or those with higher surgical risk, contributing to reduced morbidity, shorter operative time, and improved postoperative comfort. Future studies with larger samples and long‐term follow‐up may further support its adoption in clinical practice.

## CONCLUSION

The modified Neumann incision technique with a mesial relaxing cut proved to be a viable and safe approach for implant placement in the interforaminal region of the mandible in overdenture rehabilitations. The technique allowed adequate anatomical exposure, predictable access to the mental foramen, and favorable soft‐tissue healing.

Within the limits of this case series, the procedures were characterized by short surgical time, limited anesthetic requirement, and the absence of relevant postoperative complications. Healing progressed favorably, and gingival bleeding and PD values remained within clinically acceptable standards, indicating short‐term peri‐implant tissue stability.

These outcomes suggest that the technique may represent a conservative and predictable option for edentulous mandible rehabilitation. However, further controlled clinical studies with larger samples and comparative designs are necessary to confirm these findings.

## AUTHOR CONTRIBUTIONS

**Lucas Jardim da Silva**: Conceptualization; investigation; methodology; writing—original draft; data curation, software. **Laura Lourenço Morel**: Investigation; writing—original draft; methodology; data curation. **Luciana de Rezende Pinto**: Investigation; writing—original draft; methodology; writing—review and editing. **Otacílio Luiz Chagas Júnior**: Investigation; conceptualization; methodology; writing—review and editing; software. **Jamil Awad Shibli**: Conceptualization; methodology; formal analysis; writing—review and editing. **Fernanda Faot**: Conceptualization; investigation; funding acquisition; writing—review and editing; validation; project administration; supervision.

## CONFLICT OF INTEREST STATEMENT

The authors declare no conflicts of interest.

## PATIENT CONSENT FOR PUBLICATION

Written informed consent was obtained from all patients for participation in the study and for the publication of anonymized clinical data and images in this case series.

## TRIAL REGISTRATION

This clinical trial is registered at the Brazilian Registry of Clinical Trials (ReBEC) under the number RBR‐9d94bxv.
